# Complete response with early introduction of cabazitaxel in a patient with multiple lung metastases of castration-resistant prostate cancer following the early detection of metastases using liquid biopsy: a case report

**DOI:** 10.1186/s12885-019-5782-2

**Published:** 2019-06-11

**Authors:** Takeo Kosaka, Hiroshi Hongo, Mototsugu Oya

**Affiliations:** Department of Urology, Keio University School of Medicine, 35 Shinanomachi, Shinjuku-ku, Tokyo, 160-8582 Japan

**Keywords:** Cabazitaxel, Castration-resistant prostate cancer, Circulating tumor cells

## Abstract

**Background:**

Cabazitaxel (CBZ) chemotherapy for metastatic castration-resistant prostate cancer (mCRPC) is believed to be palliative because the radiological response rate is low and a durable response is rare. Here, we describe a rare case of a patient with mCRPC who was treated with CBZ chemotherapy and showed a durable radiological response and a complete biochemical response.

**Case presentation:**

A 43-year-old man with prostate cancer and metastasis of the pubic bone underwent neoadjuvant androgen deprivation and docetaxel therapy, followed by laparoscopic prostatectomy, extended lymphadenectomy, and metastatectomy in 2014. Pathological examination revealed residual adenocarcinoma in the prostate and pubic bone (pathological T stage 3b, positive surgical margin). Following the operation, he received adjuvant radiation therapy (66 Gy) to the pelvic floor. His serum prostate-specific antigen (PSA) level decreased to < 0.01 ng/mL but gradually increased following docetaxel chemotherapy. Imaging findings indicated five tiny nodules in the bilateral lungs. Biopsy specimens are difficult to obtain and might not reflect the precise extent of the disease owing to heterogeneity in patients with CRPC. Thus, we performed liquid biopsy to isolate circulating tumor cells (CTCs), and overall 156 CTCs were detected per 7.5 mL. Almost all CTCs were androgen receptor-negative in the nucleus**.** We diagnosed the five nodules as lung metastases from docetaxel-resistant CRPC with few AR-signaling-dependent cancer cells. The patient was initiated on CBZ chemotherapy (25 mg/m^2^) according to the standard protocol in August 2016, instead of using a second-generation AR-targeting agent. After 2 cycles of CBZ chemotherapy, PSA level decreased to < 0.01 ng/mL and the lung metastases completely disappeared, with a reduced CTC count of < 5. To date, the patient has been receiving intermittent CBZ chemotherapy.

**Conclusions:**

We presented a rare case of a patient with mCRPC who was successfully treated with early CBZ chemotherapy. The early detection of metastasis using liquid biopsy enabled the introduction of early CBZ chemotherapy for docetaxel-resistant mCRPC.

## Background

Cabazitaxel (CBZ) is a next-generation taxane that is indicated for the treatment of patients with metastatic castration-resistant prostate cancer (mCRPC) who were previously treated using a docetaxel-containing regimen [1]. However, CBZ chemotherapy for patients with mCRPC is believed to be palliative because the radiological response rate is low and a durable response is rare. Here, we describe a rare case of a patient with mCRPC who was treated with CBZ chemotherapy and demonstrated a durable radiological response and a complete biochemical response.

## Case presentation

**Fig. 1:**
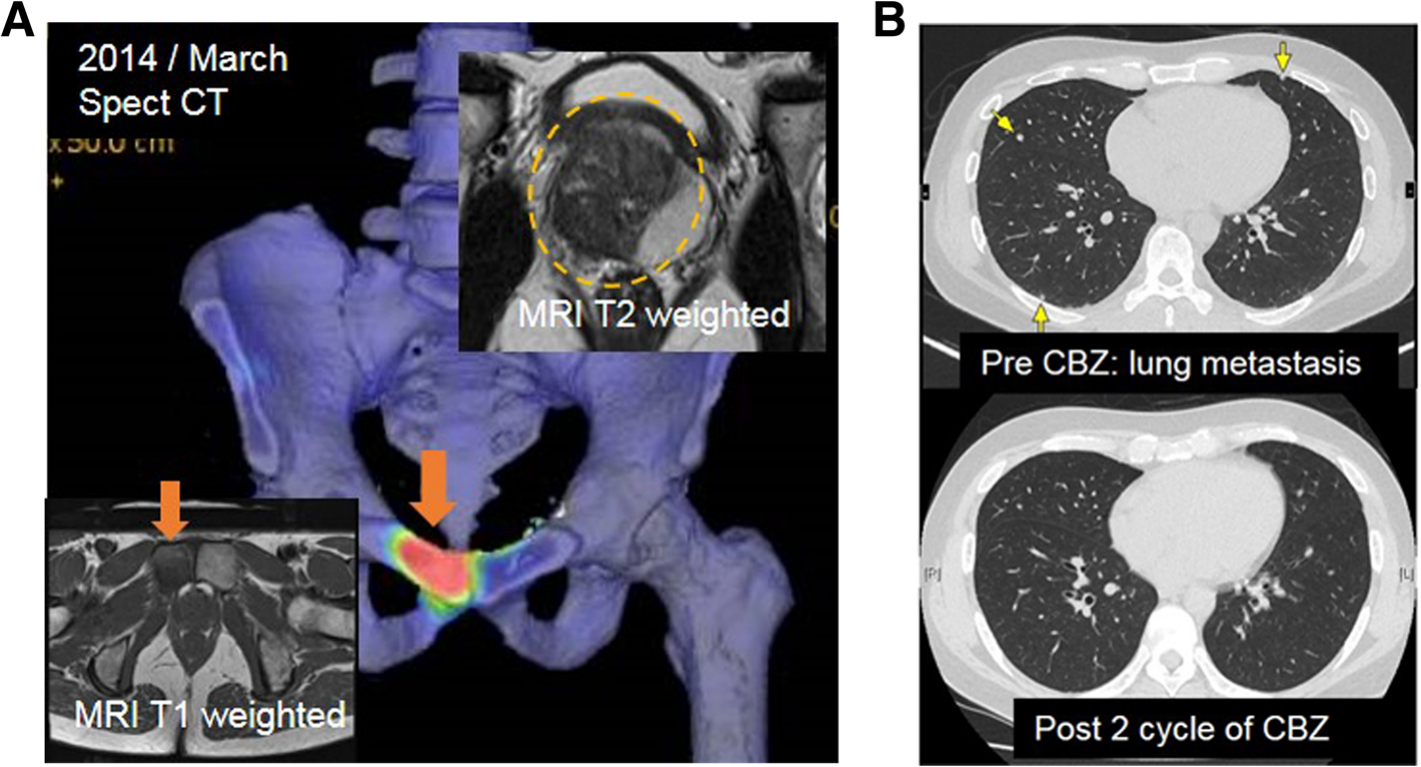
**a** Magnetic resonance imaging and bone single-photon emission computerized tomography show a prostate tumor invading the seminal vesicle and bone metastasis in the pubic bone. **b** Positron emission tomography-computed tomography shows tiny nodules in the bilateral lungs. Following 2 cycles of cabazitaxel chemotherapy, the lung metastases completely disappeared

**Fig. 2:**
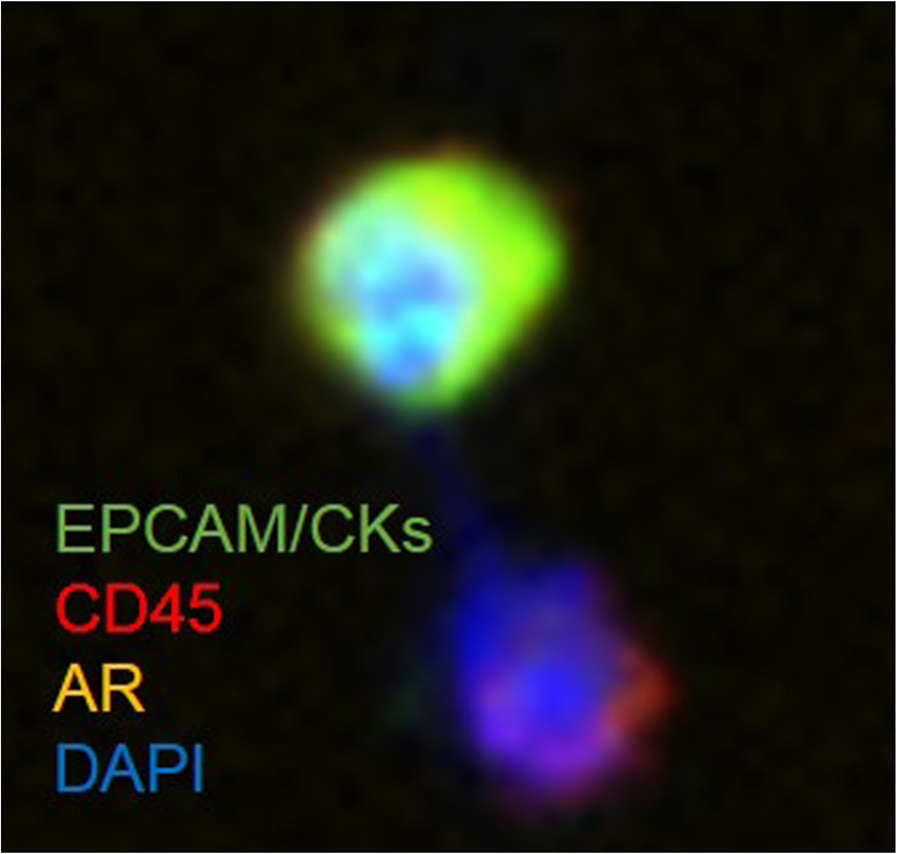
An automated circulating tumor cell (CTC) enrichment system powered by a microfluidics biochip detects CTCs that are androgen receptor-negative in the nucleus

A 43-year-old man was diagnosed with metastatic prostate cancer (Gleason score 4 + 4) in November 2013. Laboratory data showed that the prostate-specific antigen (PSA) level was 18.6 ng/mL, and digital rectal examination indicated a stony hard mass in the prostate that was suspected to be local advanced prostate cancer. Magnetic resonance imaging revealed a prostate tumor invading the seminal vesicle and a metastasis of the pubic bone (Fig. 1a). Based on these results, the patient underwent neoadjuvant androgen deprivation and docetaxel therapy, followed by laparoscopic prostatectomy, extended lymphadenenolectomy, and metastatectomy of the pubic bone in March 2014. Pathological examination revealed residual adenocarcinoma in the prostate and pubic bone (pathological T stage 3b, positive surgical margin). After the operation, he received adjuvant radiation therapy (66 Gy) to the pelvic floor. His serum PSA level decreased to < 0.01 ng/mL but gradually increased to 0.14 ng/mL. He was then re-initiated on docetaxel in December 2015, although computed tomography (CT) and bone scan did not show obvious metastatic lesions. His PSA level decreased to < 0.01 ng/mL in April 2016 after 7 cycles of docetaxel chemotherapy but slightly increased to 0.17 ng/mL in July 2016. Positron emission tomography-CT indicated five tiny nodules in the bilateral lungs (Fig. 1b). Biopsy specimens are difficult to obtain and might not reflect the precise extent of the disease owing to heterogeneity in patients with CRPC. Therefore, we performed liquid biopsy to isolate circulating tumor cells (CTCs) using the ClearCell FX System, which is an automated CTC enrichment system that is powered by a microfluidics biochip [2]. To count the CTCs isolated using this system, we performed immunostaining using the following antibodies: mouse anti-pan human keratin (C11) monoclonal antibody (mAb) (keratin 4, 5, 6, 8, 10, 13, and 18; Cell Signaling, Danvers, MA, USA), mouse anti-human cytokeratin mAb (CK3-6H5, Miltenyi Biotec GmbH, Bergisch Gladbach, Germany), mouse anti-human EpCAM (VU1D9) mAb (Cell Signaling), goat N-terminal androgen receptor (AR; N-10) polyclonal antibody (Santa Cruz Biotechnology, Santa Cruz, CA, USA), and rabbit CD45 (D9M81) mAb (Cell Signaling). Additionally, we performed nuclear staining using 4′,6-diamidino-2-phenylindole [2]. Overall, 156 CTCs were detected per 7.5 mL, and almost all CTCs were AR negative in the nucleus (Fig. 2). Therefore, we diagnosed the five tiny nodules as lung metastases from docetaxel-resistant CRPC with few AR-signaling-dependent cancer cells.

The patient was initiated on CBZ (25 mg/m^2^) according to the standard protocol in August 2016, instead of a second-generation AR-targeting agent (enzalutamide or abiraterone) [3]. Following 2 cycles of CBZ chemotherapy, the PSA level decreased to < 0.01 ng/mL and the lung metastases completely disappeared, with a reduced CTC count of < 5**.** To date, the patient has been receiving intermittent CBZ chemotherapy.

## Discussion

We experienced a patient with mCRPC who was successfully treated with CBZ chemotherapy. The patient exhibited multiple lung metastases of CRPC, and a complete response was noted with the early introduction of CBZ.

Unmet needs in patient management include the ability to monitor treatment effects and the early detection of metastasis. A common shortcoming of a CTC enrichment system is its reliance on the positive selection of CTCs with antibodies against EpCAM, suggesting the limited ability to identify CTCs with reduced EpCAM expression because of epithelial–mesenchymal transition (EMT) [4–6]. In fact, in our patient, antibodies targeting EpCAM and CD45 were only detected in 31% of CTCs, suggesting the limited ability to detect CTCs according to EpCAM. Microfluidics involves several separation methods that facilitate the manipulation of extremely small volumes of biological fluids, thereby enabling the detection of de-differentiated CRPC with EMT.

## Conclusion

We present a rare case of a patient with mCRPC who was successfully treated with early CBZ chemotherapy. The early detection of metastasis using liquid biopsy enabled the introduction of early CBZ chemotherapy for docetaxel-resistant mCRPC.

## Data Availability

Not applicable.

## Abbreviations

CBZ: Cabazitaxel
CTCs: Circulating tumor cells
EMT: Epithelial–mesenchymal transition
mCRPC: metastatic castration-resistant prostate cancer
